# Supplementary material for: Analysis of Perioperative Platelet Indices and Their Prognostic Value in Head and Neck Cancer Patients Treated with Surgery and Postoperative Radiotherapy: A Retrospective Cohort Study
Source: J Clin Med. 2019 Nov 2;8(11):1858. doi: 10.3390/jcm8111858 (PMC6912733; doi:10.3390/jcm8111858)
Supplement: Supplementary file 1 [file jcm-08-01858-s001.pdf]

Supplementary Tables

Supplementary Table 1 Preoperative platelet indices: Univariable and multivariable time-to-event analysis. Multivariable models were adjusted for TNM-stage, HPV status and smoker status.

	**Univariable**			**Multivariable**		
	HR	95% CI	p-value	HR	95% CI	p-value
**Overall survival**						
COP (per 50 G/l increase)	1.04	0.87-1.23	0.645	1.01	0.85-1.19	0.908
MPV (per 1 FL increase)	1.02	0.81-1.30	0.807	0.98	0.77-1.24	0.891
MPV/COP ratio per 1 increase	1.05	0.91-1.21	0.473	1.05	0.90-1.22	0.490
**Disease-free survival**						
COP (per 50 G/l increase)	1.02	0.84-1.24	0.812	1.00	0.82-1.23	0.944
MPV (per 1 FL increase)	1.03	0.77-1.37	0.811	1.10	0.80-1.51	0.550
MPV/COP ratio per 1 increase	1.01	0.85-1.21	0.856	1.03	0.85-1.24	0.724

Supplementary Table 2 Frequency of increase or decrease in platelet indices at day 1 and week 1 compared to the corresponding preoperative measurement. Absolute and relative frequencies are given.

		**1 day**	**1 week**
**COP**	↑	11 (10%)	79 (72%)
	↓	101 (90%)	31 (28%)
**MPV**	↑	38 (37%)	34 (33%)
	↓	65 (63%)	69 (67%)
**MPV/COP ratio**	↑	94 (91%)	28 (27%)
	↓	9 (9%)	75 (73%)

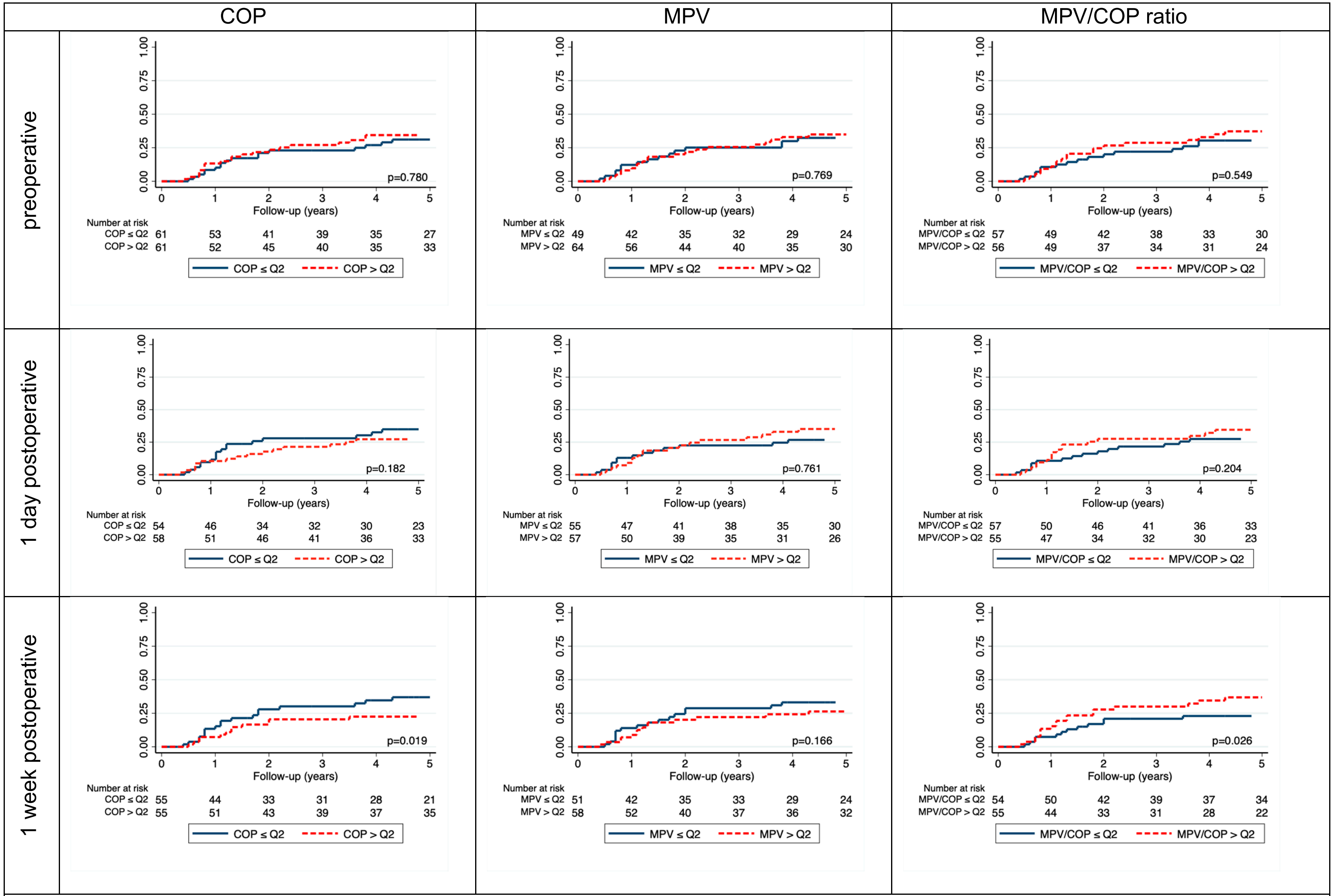

Suppl. Fig. 1 Kaplan-Maier failure function according to preoperative (top row) and postoperative (bottom rows) platelet indices for disease-free survival (DFS).
